# Political Competence and Evidence-Informed Policy Engagement Among Nurses and Nursing Students in Croatia: A Cross-Sectional Study

**DOI:** 10.3390/nursrep15120448

**Published:** 2025-12-14

**Authors:** Nikolina Brešić, Mate Car

**Affiliations:** 1 Department of Nursing, Catholic University of Croatia, 10000 Zagreb, Croatia; 2 Department of Primary Care and Public Health, Imperial College London, London SW7 2AZ, UK

**Keywords:** political competence, nursing leadership, evidence-informed policy, nurse education, health policy engagement, empowerment, Croatia

## Abstract

**Background**: Nurses have unique insights into health systems yet remain underrepresented in policy processes. Strengthening political competence is widely regarded as important for evidence-informed nursing leadership. **Objectives**: To assess self-perceived political competence and policy participation among nurses and nursing students in Croatia. **Methods**: We conducted a cross-sectional online survey using open, non-probability recruitment. Of 447 individuals who opened the survey link, 440 provided responses and 390 completed all 19 competence items measuring political competence across three domains (attitudes, knowledge/evidence, and skills). Open recruitment precluded calculation of a conventional response rate. A 19-item self-report questionnaire measured political competence across three domains (attitudes, knowledge/evidence and skills); internal consistency was α = 0.91, although the Croatian version has not been fully validated. Data were analyzed using descriptive statistics, binomial tests and ordinal logistic regression on the complete-case sample. **Results**: Most respondents endorsed nurses’ policy involvement, the importance of scientific evidence and nursing’s holistic perspective. **Conclusions**: Nurses and nursing students reported strong conceptual support for a policy role but only moderate confidence in influencing policy. These cross-sectional findings highlight a gap between conceptual support and personal confidence that may be addressable through structured educational and institutional support; the impact of such initiatives on policy influence should be examined in future longitudinal or interventional studies.

## 1. Introduction

Nurses constitute the largest professional group in healthcare and play an essential role in sustaining population health. Their close contact with patients provides unique insight into the functioning of health systems and the social determinants of health. Despite this, their potential to influence health policy remains underutilized. Studies from diverse health systems describe that nurses are frequently positioned primarily as implementers rather than contributors to policy formulation, especially in hierarchical or physician-dominated contexts [1,2,3,4].

Political competence—defined as the knowledge, attitudes, and skills required to engage effectively in policy and governance—has therefore become a critical domain of professional development in nursing [5,6,7]. It combines political literacy, ethical reasoning, communication, and advocacy abilities that enable nurses to act as credible policy partners. Strengthening these competencies is linked to greater professional autonomy, improved health outcomes, and stronger leadership within multidisciplinary teams [8,9].

In this study, we conceptualized political competence as a multidimensional construct comprising three domains: (1) attitudes and normative beliefs about the role of nursing in health policy (e.g., whether nurses should participate in policy formulation and whether nursing’s holistic vision makes it central to policy change); (2) political knowledge and evidence orientation, reflected in recognizing the importance of scientific evidence for nursing practice and health-policy decisions; and (3) self-perceived political skills and self-efficacy, understood as confidence in one’s ability to use critical thinking, formulate proposals for change, and influence health policy and the perception of nursing within the health-care team. Sociodemographic and professional characteristics (age, years of experience, and membership in professional or civic associations) were treated as contextual factors that may shape these domains of political competence and, in turn, nurses’ readiness to participate in health-policy development.

Although international strategies emphasize the inclusion of nurses in policy processes, the development of political competence remains uneven across regions. Evidence from Western Europe and North America shows that nurses with higher policy literacy and civic engagement are more likely to contribute to patient-safety initiatives and health-system reform [10]. In contrast, studies from Central and Eastern Europe highlight persistent structural challenges, including limited professional representation and high levels of unfinished nursing care, which reflect systemic barriers to nursing advocacy and policy participation [11]. During the European Union accession period, Croatia and other new member states experienced what De Raeve described as a “policy window opportunity” for nursing leadership and professional advocacy—an opportunity that remains only partially realized [12].

Recent reviews have synthesized strategies for developing nurses’ policy competence and participation but also highlight important limitations. A systematic review of seven educational interventions designed to encourage nurses’ participation in health policy found that such interventions can increase self-reported competence, but that the evidence base is small and heterogeneous [13]. An evidence-based commentary on strategies for policy-competence education similarly argues that, although numerous educational proposals exist, their implementation and evaluation remain uneven and context-dependent, and calls for more systematic, context-sensitive programs [14]. A recent scoping review of 32 studies on political competency among nurses and nursing students reported that research focuses mostly on political participation, while research into interaction skills and conceptual clarity is weak and validated instruments remain scarce and are context-specific [15]. In parallel, a qualitative meta-synthesis of nurses’ political participation identified low political interest and competence, insufficient political education, restrictive organizational cultures, and weak professional organizations as key barriers, indicating that educational interventions alone are unlikely to overcome structural constraints [16]. A broader literature review on nursing and midwifery’s influence on health policy further points to power imbalances, gendered dynamics, and limited organizational support, and calls for multi-level strategies that combine education with leadership development and strengthened professional associations [17]. Together, these findings suggest that policy-oriented education can enhance nurses’ knowledge and self-perceived readiness to engage in policy, but that its impact remains constrained by conceptual, methodological, and systemic limitations, particularly in under-studied settings such as Central and South-Eastern Europe.

Developing political competence among nurses is therefore not only an educational priority but also a matter of workforce sustainability and ethical responsibility. Greater engagement in policy can address long-standing issues such as staffing shortages, burnout, and inequitable resource allocation [10]. Building nurses’ capacity to use evidence for advocacy aligns with the World Health Organization’s call for “evidence-informed leadership” in nursing and midwifery [18]. Such engagement enhances the visibility of nursing expertise and supports democratic, transparent health-system governance.

Empirical work in this field has demonstrated that political self-efficacy—the belief in one’s ability to influence policy—is a strong predictor of actual participation [19]. Studies by Cervera-Gasch et al. and Han and Kim highlight that educational exposure and active membership in professional or civic associations are associated with higher scores on these dimensions [19,20]. Building on this work, Han et al. recently developed and psychometrically evaluated the Political Competence Scale for Nurses (PCS-N), providing one of the first dedicated instruments with formal validity evidence, albeit in the Korean context [19].

The available empirical and review evidence is drawn predominantly from settings outside Central and South-Eastern Europe and, to our knowledge, neither the above reviews nor our targeted search of Croatian and regional literature identified any quantitative studies assessing nurses’ political competencies or self-perceived policy confidence in Croatia. A brief targeted search of major international databases and Croatian nursing journals (to August 2025) found no prior empirical studies on nurses’ political competence or policy participation in Croatia. By surveying nurses and nursing students in the Croatian context, this study contributes to addressing this gap and adds region-specific data on perceived political competencies, barriers, and opportunities for policy engagement.

Overall, the literature points to three unresolved gaps: (1) a mismatch between nurses’ strong attitudinal support for policy engagement and their inconsistent political self-efficacy; (2) mixed and often modest effects of educational interventions on actual policy participation; and (3) an absence of quantitative evidence from Central and South-Eastern Europe, including Croatia. Addressing these gaps requires context-specific empirical data. Our study contributes to this need by examining self-perceived political competence and policy participation among nurses and nursing students in Croatia.

This study therefore aimed to assess self-perceived political competence and participation in health-policy processes among nurses and nursing students in Croatia. Specifically, we sought to describe attitudes toward nurses’ involvement in health policy, knowledge and evidence orientation, and self-reported policy-related skills, and to examine whether age, years of work experience, gender, and membership in professional or civic associations are associated with political self-efficacy. By providing quantitative, context-specific data from an under-studied Central and South-Eastern European setting, this study addresses the lack of empirical evidence on nurses’ political readiness in Croatia.

## 2. Materials and Methods

### 2.1. Study Design and Setting

A cross-sectional online survey was conducted among nurses, and nursing students in Croatia between February and June 2025. The study followed the Checklist for Reporting Results of Internet E-Surveys (CHERRIES) and STROBE guidelines for observational research. The survey link was distributed through university mailing lists, social networks, and QR codes displayed at the Catholic University of Croatia. This was a non-probability convenience sample, and the survey link was disseminated widely via university and professional networks; no sampling frame or response rate could be calculated. The survey did not intentionally include a direct item distinguishing students from employed nurses. This design choice reflected the overlap between study and employment roles in the Croatian context and aimed to reduce questionnaire length and redundancy. Instead, age and years of work experience were recorded as proxies for respondents’ position along the training-to-practice continuum. Participation was voluntary and anonymous; informed consent was required.

### 2.2. Participants and Eligibility Criteria

Eligible participants were:

- Registered nurses, or nursing students,
- Residing in Croatia,
- Aged ≥18 years.

### 2.3. Instrument

Data were collected using a structured questionnaire previously developed by Cervera-Gasch et al. [20] and adapted for the Croatian context with the authors’ permission. The Croatian version was produced through forward- and back-translation by two independent translators, followed by a pilot test with 10 volunteers (nurses and nursing students recruited separately from the main study channels). None of the pilot participants were included in the final dataset. The pilot confirmed overall clarity and flow; only minor wording adjustments were made, and no items were added or removed before launching the full survey.

The questionnaire items were grouped into three substantive domains of political competence. The attitudes and normative beliefs domain included items on support for nurses as actors in health-policy-making, the perceived influence of working conditions on nurses’ policy participation, and the view that nursing’s holistic, person-centered vision makes it essential for changing health policy. The political knowledge and evidence-orientation domain comprised items on the perceived importance of scientific evidence for health-policy decisions and for strengthening nursing as a profession, as well as the perceived ability to use scientific evidence to initiate policy changes in practice. The self-perceived skills and self-efficacy domain included items on critical-thinking skills, perceived ability to formulate proposals for policy change, perceived ability to influence health policy, and confidence in influencing how other members of the health-care team view nursing.

For the directed hypotheses, we selected four sentinel items from these domains as primary outcomes: recognition of the influence of working conditions on nurses’ policy participation (H1, attitudes), recognition of scientific evidence as a fundamental pillar of the profession (H2, knowledge/evidence orientation), viewing nursing’s holistic perspective as essential for health-policy change (H3, attitudes), and self-rated ability to formulate proposals and influence policy (H4, skills/self-efficacy). We hypothesized that a majority of respondents would endorse the importance of working conditions, scientific evidence, and the holistic perspective (H1–H3), and that respondents with fewer than five years of experience would report higher political self-efficacy than those with longer experience (H4).

The final instrument comprised 5 demographic questions and 19 Likert-scale items covering three domains derived from the original instrument (Attitudes, Knowledge/Evidence, and Skills) [20]. Some closely related domains (knowledge and attitudes) were combined for brevity in this adapted version, but all key content areas were preserved. Each item was rated on a five-point Likert scale (1 = strongly disagree to 5 = strongly agree). Sociodemographic variables included gender, age, years of professional experience, and membership or active participation in professional or civic associations (including a follow-up question on active role for members).

Internal consistency in our sample was excellent for the total 19-item set (Cronbach’s α = 0.91) and acceptable to good at the domain level (Attitudes α = 0.69; Knowledge/Evidence α = 0.73; Skills α = 0.84). Domain-level α coefficients and item-level descriptive statistics are summarized in Supplementary Table S1. We used these indices as descriptive diagnostics of the adapted instrument’s internal consistency; the study was not designed as a full psychometric re-validation.

Consistent with this structure, the selection of sentinel items for hypothesis testing and the interpretation of outcomes followed the three-domain political-competence framework defined in the Introduction, in which attitudes/normative beliefs, knowledge and evidence orientation, and skills/self-efficacy represent complementary dimensions of nurses’ political readiness.

### 2.4. Data Collection Procedure

Participants accessed the survey through a secure LimeSurvey platform. Before proceeding, each respondent read an electronic information sheet describing study aims, anonymity, and withdrawal rights; only those who clicked “I agree” could continue. Duplicate submissions were prevented by single-use links and IP screening. The estimated completion time was nine minutes.

### 2.5. Ethical Considerations

The study was approved by the Ethics Committee of the Catholic University of Croatia (KLASA:602-04/25—11/01, UBROJ:251-498-03-02-25-2, 27 January 2025). Participation was voluntary and confidential, and informed consent was required.

### 2.6. Data Analysis

This was an exploratory, non-probability online survey without a defined sampling frame or reliable prior estimates of effect sizes. We therefore did not perform a formal a priori sample size calculation; instead, we aimed to recruit as many eligible nurses and nursing students as possible during the data-collection period. The complete-case analytic sample for inferential analyses comprised N = 390 respondents who answered all 19 competency items.

Missing data and sample size: For descriptive analyses we used all available responses for each item (pairwise deletion). Thus, N = 440 for overall participant demographics, and item-level results use the actual respondents per item (e.g., n = 413 answered the first section of competency items). For all hypothesis tests and regression analyses, we used a single complete-case analytic sample restricted to the 390 respondents who completed all 19 competency items.

Given the low proportion of missing competency items and the exploratory aim, we did not impute missing values; instead we used a transparent complete-case analytic set for all hypothesis tests and regression models. However, neither pairwise deletion nor complete-case analysis can correct for bias if missingness is not random. In particular, if respondents with lower political confidence or weaker knowledge were more likely to skip items, agreement estimates could be somewhat inflated and regression coefficients attenuated. Because we did not have auxiliary variables to model missingness, we could not perform a formal sensitivity analysis, and thus all point estimates should be interpreted with this limitation in mind.

In exploratory sensitivity analyses, we also summarized key outcomes stratified by age group and membership in professional or civic associations (Table S4).

For descriptive purposes, Likert responses were coded from 1 (“strongly disagree”) to 5 (“strongly agree”). Agreement was defined a priori as scores 4–5, reflecting explicit endorsement, whereas 3 indicated neutrality/uncertainty. For three pre-specified “sentinel” items we estimated the proportion agreeing and tested against a 50% benchmark using two-sided exact binomial tests, reporting exact 95% confidence intervals. To respect the ordinal nature of the scale, we additionally report medians (IQR) and full response distributions and conducted Wilcoxon signed rank tests versus a neutral value of 3 as a sensitivity analysis (Supplement Table S3).

We pre-specified four item-level hypotheses using sentinel statements that represent core facets of the constructs (working conditions; evidence salience; holistic perspective; self-assessed policy confidence). We did not aggregate items into new domain scores for confirmatory tests because the Croatian adaptation was not designed or powered as a full validation study. Domain-level internal consistency (Cronbach’s α) and item distributions are reported descriptively (Supplementary Table S1); inferential tests target the pre-specified items.

Statistical tests: We used exact binomial tests (two-sided) to evaluate whether the proportion agreeing with key statements exceeded 50% (null hypothesis 0.50). The exact binomial test was chosen over a one-sample chi-square because it provides an exact *p*-value and confidence interval without large-sample assumptions, appropriate here since we tested predefined 50% thresholds.

For the hypothesis comparing less-experienced vs. more-experienced nurses, we employed a one-sided Fisher’s exact test (H1: <5 years’ experience group shows a higher % agreement) due to small subgroup sizes. To assess predictors of self-rated policy confidence (an ordinal outcome), we used ordinal logistic regression (proportional odds model) on the complete-case sample (N = 390).

For Likert-scale items (1–5), we treated responses as ordinal and summarized them using medians and interquartile ranges (IQR), together with the full distribution of responses across categories. For descriptive purposes and to test hypotheses specified as “>50% agreement”, we additionally reported the proportion of respondents who selected 4 (“agree”) or 5 (“strongly agree”) and applied exact binomial tests to these proportions.

Ordinal logistic regression was chosen instead of linear regression or multinomial logistic regression to appropriately model the ordered nature of the Likert-scale outcome without assuming equal intervals. We report adjusted odds ratios (aOR) with 95% CI. Proportional-odds assumptions were assessed using the Brant test. The global test indicated a violation driven by membership in professional or civic associations, so we fitted a generalized ordered logit sensitivity analysis (VGAM). The direction and significance of key predictors were unchanged. Model fit was checked via the Brant test for proportional odds assumption and variance inflation factors (VIF) for multicollinearity. Clopper–Pearson exact CIs were calculated for all proportions. A two-tailed α = 0.05 defined statistical significance.

Covariates for the ordinal logistic regression were pre-specified a priori based on prior studies of nurses’ political engagement and on variables available for all respondents. We included years of nursing work experience and age at professional start to capture career stage and cohort effects, membership in professional or civic associations (yes/no) as an indicator of structural engagement with professional or civic organizations, and gender as a key sociodemographic characteristic in a highly feminized profession. To maintain a parsimonious model and avoid overfitting, no additional predictors were entered.

Information on education level and detailed workplace setting was not collected in this survey, so we could not adjust for these potential confounders.

All analyses were conducted in R (version 4.5.1; R Foundation for Statistical Computing, Vienna, Austria) using the tidyverse, psych, and gtsummary packages.

## 3. Results

### 3.1. Participant Characteristics

A total of 447 participants opened the survey link, of whom 440 provided responses that were included in the analysis (at a minimum, answered the demographic section; see Table 1). Of these, 413 proceeded to answer at least the first section of policy-related questions, and 390 (88.6% of all respondents) completed all 19 competency items. We retained partially completed questionnaires for which substantial data were available, using pairwise deletion for descriptive analysis as noted in Methods. Thus, the sample size varies by analysis: for example, Table 2 reports item statistics for N = 413 respondents in the Attitudes section, whereas regression in Table 3 and hypothesis tests in Table 4 use N = 390. Participants were 84.1% female (370/440). The largest age group was 18–24 years (31.4%, n = 138), followed by 25–35 years (26.8%, n = 118), highlighting a younger, early-career segment within the sample. Mean professional experience was 11.3 years (SD = 11.1; median = 7; IQR = 1–20). Just over half (52.3%) reported membership in a professional or civic association, and 17% identified as active members. Membership in professional or civic associations increased steadily with age—from 33% among participants aged 18–24 years to 71% among those older than 50. Note: A conventional response rate could not be calculated, as recruitment used open channels without individual view tracking (many potential participants may have seen the invitation; we only know 447 clicked the survey and 440 provided usable data); thus, we cannot estimate the proportion of those exposed who participated.

**Figure 1:**
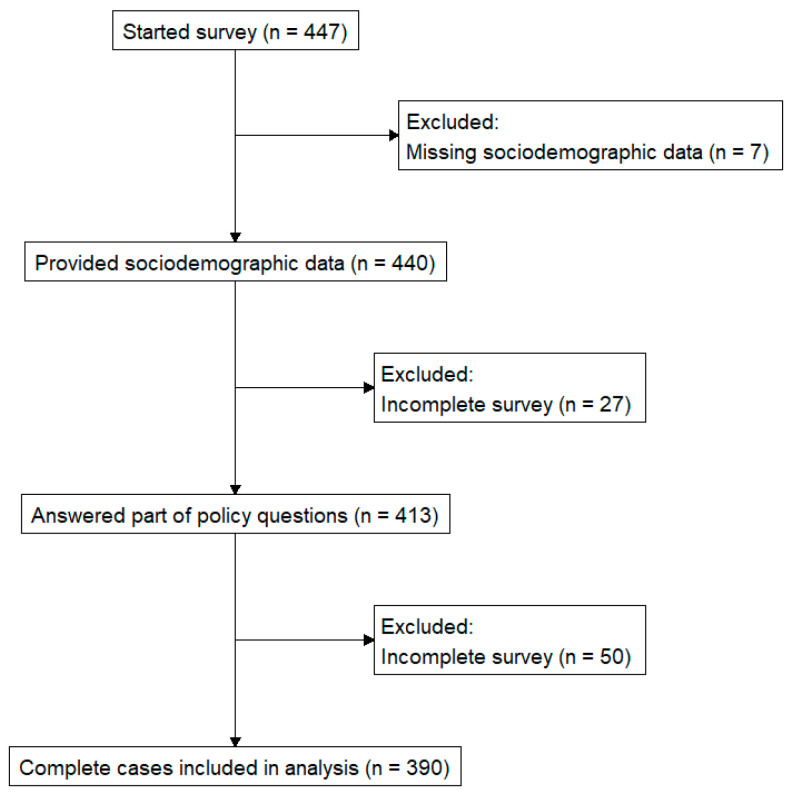
Study flowchart of participant recruitment, exclusions, and final analytic sample.

Figure 1 presents the study design flowchart, outlining participant inclusion and exclusion.

**Table 1:** Sociodemographic characteristics of participants (N = 440).

Characteristic	N = 440
Gender	
Male	70.0 (15.9%)
Female	370.0 (84.1%)
Age group (years)	
18–24	138.0 (31.4%)
25–35	118.0 (26.8%)
36–45	93.0 (21.1%)
46–50	56.0 (12.7%)
50+	35.0 (8.0%)
Mean age (years)	33.6 ± 11.2; 30.0 (21.0–40.0)
Work experience (years)	11.3 ± 11.1; 7.0 (1.0–20.0)
Age at professional start (years)	22.7 ± 4.8; 21.0 (20.0–25.0)
Member in professional or civic associations	230.0 (52.3%)

Values are presented as n (%), or mean ± SD; median (Q1–Q3) for continuous variables. Age at start capped at minimum 18 years to ensure plausible entry age. 0 years’ experience = 70 participants.

### 3.2. Attitudes Toward Nursing and Policy Participation

Respondents demonstrated highly positive views about nurses’ engagement in health policy.

- 84% supported the statement that nurses can be agents of change in health policy.
- 75% agreed that nurses should participate directly in political decisions.
- 87% believed that nursing should propose changes to improve the health system.
- Perceived knowledge of nursing leadership was moderate (mean = 3.12, SD = 1.08).

Hypothesis tests are item-level (exact binomial/Fisher) aligned to the four sentinel statements.

**Table 2:** Attitudes and self-assessed knowledge (Likert 1–5) (N = 413).

Statement	Mean (SD)	Median (Q1–Q3)	% Agree (4–5)
Nurses can create change in health policy	4.31 (0.97)	5.00 (4.00–5.00)	84%
Nurses should participate in political decision-making	4.14 (1.09)	5.00 (4.00–5.00)	75%
Nursing should propose reforms in health care	4.51 (0.92)	5.00 (4.00–5.00)	87%
I have sufficient knowledge of nursing leadership	3.11 (1.09)	3.00 (2.00–4.00)	38%

Values represent Mean (SD) on a 1–5 Likert scale, Median (Q1–Q3 interval) and % Agree (scores 4–5).

Sensitivity analyses using the full 5-point scale (Wilcoxon signed-rank tests against a neutral median of 3) yielded the same pattern of conclusions as the binomial ≥ 4 tests (Table S3).

Due to missing data patterns in the original LimeSurvey export, four attitude items had N = 413 while the remaining 15 items had N = 390; full distributions are provided in Supplementary Table S2.

### 3.3. Perceived Competence and Confidence

The adapted instrument showed acceptable internal consistency across domains (Attitudes α ≈ 0.69; Knowledge/Evidence α ≈ 0.73; Skills α ≈ 0.84; Supplementary Table S1), providing support for using these item sets descriptively in this sample.

Respondents expressed confidence in applying critical thinking (69% agreed they could use it to improve practice), yet less than half (47%) felt able to formulate specific policy proposals.

In multivariable ordinal logistic regression (N = 390), age at professional start was inversely associated with confidence (aOR 0.94; 95% CI 0.91–0.98; *p* = 0.005). Experience (aOR 1.01; 1.00–1.03; *p* = 0.162), membership (aOR 0.91; 0.63–1.31; *p* = 0.604), and gender (aOR 1.00; 0.60–1.65; *p* = 0.986) were not statistically significant. Adjusted odds ratios are presented in Supplementary Figure S1.

Diagnostics and robustness: the Brant test indicated a proportional-odds violation driven by membership in professional or civic associations, while age at professional start, experience, and gender met the assumption. We therefore fitted a generalized ordered-logit model (VGAM) allowing membership effects to vary across thresholds (Supplementary Table S5); estimates for age at professional start, experience, and gender were consistent with those in the proportional-odds model, and the interpretation of membership did not materially change. All VIF were <2; pseudo-R^2^ = 0.09; likelihood-ratio *p* < 0.001. The model for perceived ability to formulate proposals for policy change showed statistically significant associations for age at professional start, while the overall explanatory power was modest (McFadden pseudo-R^2^ = 0.09), which is typical for models predicting self-reported attitudes and suggests that additional unmeasured factors likely influence political self-efficacy.

In the adjusted model (N = 390) shown in Table 3, lower age at professional start was significantly associated with higher confidence, while work experience, gender, and membership were not significant.

**Table 3:** Predictors of self-assessed policy confidence (ordinal logistic regression), (N = 390).

Variable	aOR (95% CI)	*p*-Value
Work experience (per year)	1.01 (1.00–1.03)	0.162
Age at professional start (per year)	0.94 (0.91–0.98)	0.005
Association membership (yes vs. no)	0.91 (0.63–1.31)	0.604
Gender (female vs. male)	1.00 (0.60–1.65)	0.986

Footnote: aOR = adjusted odds ratio; CI = confidence interval; model adjusted for experience, age at start, membership, and gender. Outcome coded 1–5 (higher = greater self-assessed policy confidence). Missing values were excluded listwise.

In a generalized ordered-logit sensitivity analysis allowing membership effects to vary across thresholds (Table S5), estimates for age at professional start, experience, and gender were consistent with those in the proportional-odds model. Membership effects varied by threshold but did not materially change the interpretation.

### 3.4. Evidence-Informed Practice and Hypothesis Testing

Recognition of evidence-based policymaking was strong across the sample:

- 78.5% agreed that working conditions affect nurses’ policy participation (*p* < 0.001 vs. 50%).
- 72.3% agreed that scientific evidence is key to nursing policy (*p* < 0.001).
- 80.5% agreed that nursing’s holistic perspective is essential for policy reform (*p* < 0.001).

Less-experienced respondents showed 51.8% agreement (71/137; 95% CI 43.1–60.4) vs. 44.7% among those with ≥5 years (113/253; 95% CI 38.4–51.0); this did not reach significance in the prespecified one-sided Fisher test (*p* = 0.106).

**Table 4:** Hypothesis tests for agreement and experience-related confidence (exact tests) (N = 390).

Hypothesis	% Agree (≥4)	95% CI	*p*-Value (Exact)	Result
**H1:** *Working conditions affect nurses’ policy participation*	78.5% (306/390)	74.0–82.4%	<0.001	Confirmed
**H2:** *Scientific evidence is key to nursing policy*	72.3% (282/390)	67.6–76.7%	<0.001	Confirmed
**H3:** *Nursing’s holistic perspective is essential for reform*	80.5% (314/390)	76.2–84.3%	<0.001	Confirmed
**H4:** *Less-experienced nurses (<5 yrs) show higher confidence*	<5: 51.8% (71/137) ≥5: 44.7% (113/253)	43.1–60.4% 38.4–51.0%	0.106	Not confirmed

Notes: For H1–H3, two-sided exact binomial tests vs. 50% agreement; 95% CIs are Clopper–Pearson. For H4 (<5 vs. ≥5 years), group-specific %Agree are shown with Clopper–Pearson 95% CIs; a one-sided Fisher’s exact test evaluates the difference (H1: <5 years has higher %Agree). Analytic set: complete-case N = 390. Likert coded 1–5; %Agree counts scores ≥ 4.

Stratified %Agree results for two key outcomes (ability to propose policy changes; influence of working conditions) are shown in Supplementary Table S4. Patterns for perceived ability to propose policy changes were similar, with modest variation by age and association membership.

The generalized ordered-logit model, relaxing the proportional-odds assumption for association membership, yielded results consistent with the main ordinal logistic regression. Age at professional start remained the only statistically significant predictor of higher policy confidence (aOR = 0.95, 95% CI 0.91–0.99), while work experience, gender and membership effects were not materially altered (Supplementary Table S5). Allowing non-parallel odds for membership revealed substantial threshold-specific variation—membership was associated with a markedly lower likelihood of selecting the lowest response categories (e.g., aOR = 15.42 for P (Y ≥ 2 vs. 1)), but not with consistently higher confidence across the upper thresholds (aOR = 0.91 for P (Y ≥ 4 vs. 1–3)). These findings confirm that the violation of proportional odds for membership does not change the study’s substantive conclusions.

### 3.5. Key Takeaways

Findings reveal broad conceptual support for nurses’ policy engagement and strong endorsement of evidence as a foundation for decision-making. However, personal confidence remains uneven, especially among older and more experienced participants, suggesting cultural and organizational barriers rather than attitudinal resistance. Results were consistent across sensitivity analyses and point to an educational and institutional opportunity to explore integrating political competence and evidence-informed advocacy throughout nursing curricula and professional pathways.

## 4. Discussion

The Discussion is organized around three guiding questions: how these findings compare with existing evidence; what they may suggest for nursing education and professional development; and how they inform health-policy processes and system-level governance in Croatia.

This study is among the first to explore nurses’ and nursing students’ self-perceived political competence and participation in health policy in Croatia. The findings reveal strong conceptual endorsement of nursing’s role in policy development, high recognition of the importance of evidence-informed practice, and moderate self-confidence to act as policy advocates. These results are consistent with the growing international consensus that nurses see themselves as potential change agents but often lack the structural and educational support required to translate intention into influence. As shown in the multivariable analysis (Table 3), age at professional start was the only covariate independently associated with political self-efficacy, whereas work experience, membership in professional or civic associations and gender were not statistically significant. The limited explanatory power of this model suggests that other individual and organizational factors not captured in this study are likely to be important. Given the cross-sectional design and the use of a self-selected non-probability sample, the findings are best interpreted as descriptive signals within this sample rather than precise estimates of competency levels in the national nursing workforce.

### 4.1. How Do These Findings Compare with Existing Evidence?

The overall positive orientation toward nurses’ participation in policymaking aligns with studies from Spain [20] and the Republic of Korea [19], which similarly reported high attitudinal readiness but limited practical engagement. The Croatian findings also echo data from Western Europe, where nurses endorse advocacy but cite time constraints and organizational hierarchies as barriers [10,21]. Taken together, these patterns position the Croatian findings within the broader international evidence base, indicating both convergence in attitudinal readiness and divergence in confidence-related dimensions.

Distinctly, the inverse association between age at professional start and political confidence observed here contrasts with reports from mature professional systems, where seniority predicts stronger advocacy [22]. This suggests that in transitional health systems such as Croatia’s, experience does not necessarily translate into influence—a pattern that could reflect structural factors described in prior literature, such as predominantly clinically oriented managerial pathways, although our data cannot test this explanation.

The negative association between membership in professional or civic associations and self-confidence was unexpected. One plausible explanation is that membership may reflect passive affiliation rather than active participation, or that organizational structures emphasize continuing education and licensure rather than policy training. Similar findings have been reported in Central European contexts where professional associations are still developing their advocacy mandates [11].

### 4.2. What Are the Possible Suggestions for Nursing Education and Professional Development

The generational gradient in political confidence is consistent with the idea that curricular reform might play a role, although this cannot be inferred from cross-sectional data. Younger nurses and students appear more open to policy engagement, suggesting that differences in exposure to leadership and health-system content during education may be associated with variation in professional identity. Integrating policy literacy, evidence use, and advocacy skills within nursing education has been proposed in prior literature as a way to support this momentum. These findings underscore the relevance of educational strategies that address both conceptual understanding and the confidence required to apply political competence in practice.

For example, nursing curricula in Croatia could include dedicated health policy courses or modules—an area currently underrepresented—to build literacy in legislative processes and advocacy techniques. Integrating policy literacy, evidence use, and advocacy skills within undergraduate and graduate programs (through coursework and practical projects) could reinforce this momentum. Bridging this gap likely involves both educational and institutional strategies: embedding critical appraisal exercises in coursework, offering students and staff mentorship in translating research into policy, and ensuring that nurse managers model data-driven decision-making. Such efforts are consistent with the World Health Organization’s Global Strategic Directions for Nursing and Midwifery 2021–2025, which emphasize evidence-informed leadership as a prerequisite for sustainable health-system governance [18].

### 4.3. What Are the Implications for Health-Policy Processes and System-Level Governance

From a system perspective, these findings suggest that nurses and nursing students in Croatia may be conceptually ready for greater policy involvement but remain structurally constrained. Policymakers and healthcare leaders could consider formal mechanisms to include nurses in decision-making—for example, appointing nursing representatives to hospital boards, health ministry committees, and regional health councils. Strengthening the capacity and mandate of national nursing associations for collective advocacy is also critical. The observed optimism among younger nurses represents a strategic “policy window” [12] that could be leveraged to modernize leadership pipelines and ensure that policy formulation reflects frontline realities.

Engagement should not be limited to health-sector governance; nurses can also contribute to intersectoral issues such as aging, chronic disease prevention, and workforce planning. In summary, targeted educational reforms (updating curricula) and policy changes (inclusive governance structures) may help convert nurses’ demonstrated enthusiasm into tangible influence. Investing in nursing leadership training may therefore contribute to health-system resilience and democratic policy processes. Framing these results within Croatia’s governance context suggests that strengthening political competence may support more consistent engagement of nurses in policy dialogue, even though this study cannot infer causal relationships.

Integrating the attitudinal, knowledge, and self-efficacy findings indicates that strong conceptual endorsement of nurses’ policy roles does not necessarily translate into uniform personal confidence, particularly among older and more experienced participants.

### 4.4. Strengths and Limitations

A key strength of this study is the relatively large sample (N = 440) encompassing both employed nurses and students, which permits an indicative cross-generational perspective on political competence. However, the sample is not demographically balanced—women and younger respondents are overrepresented—which limits the diversity of perspectives and should be considered when interpreting the findings. The use of a previously developed survey instrument (adapted from prior research) and our transparent reporting of recruitment, missing-data handling, and analysis enhance the credibility of the findings.

Important limitations must be acknowledged. First, data are self-reported, which can introduce social desirability bias and may inflate positive responses. Second, the cross-sectional design limits causal inference—we can only report associations, not determine directionality. Third, the sample contains a substantial early-career subgroup: around one-sixth of respondents reported 0 years of nursing work experience, and almost one-third were aged 18–24 years. This composition may reflect generational differences in optimism and self-efficacy and could overestimate political confidence relative to the employed nursing workforce. Our cross-sectional design cannot disentangle age, cohort, and student status effects. In addition, we did not collect a direct measure of professional role (student vs. employed nurse), and in the Croatian context some respondents are likely to combine work and study. Our use of age and work experience as proxies cannot distinguish pure students from mixed work–study roles, so we cannot provide precise role-specific estimates. The findings should therefore be interpreted as descriptive of this mixed nursing workforce and training pipeline, rather than of any particular occupational stratum.

We attempted to mitigate this by broad outreach, but the sample may not perfectly represent all Croatian nurses. Additionally, our handling of missing data via pairwise deletion for descriptives and a complete-case analytic set for inferential analyses could introduce bias if the likelihood of item non-response was related to unobserved factors; we did not perform multiple imputation, which would require further modelling assumptions for ordinal Likert items. These decisions imply that the exact proportions and regression coefficients should be interpreted with caution, as missing-item patterns—though limited—may slightly shift point estimates in either direction. The proportional-odds assumption did not hold for membership in professional or civic associations; however, a generalized-ordered-logit sensitivity analysis yielded similar conclusions. Despite these limitations, we believe the study provides valuable insights. The sample size for the regression analyses (N = 390 complete cases) was sufficient for stable estimation of the main age-related association but may have limited power to detect smaller effects, particularly for gender and membership in professional or civic associations. Non-significant predictors in the ordinal model should therefore be interpreted cautiously as reflecting limited sensitivity to modest associations in this exploratory, non-probability sample.

Fourth, the open online, convenience recruitment strategy via academic and professional networks likely over-represents nurses and students who are more digitally connected and potentially more motivated or interested in policy issues. Combined with the high proportion of young and early-career respondents, this may overstate attitudinal support and self-reported political confidence compared with the broader nursing workforce in Croatia. Because no sampling frame or denominator was available, we could not quantify the magnitude of this self-selection bias or compute a response rate; our findings should therefore be interpreted as descriptive of this self-selected sample rather than as population-level estimates. Because we did not record specific recruitment channels, we could not account for potential clustering of responses within institutions or networks; this may lead to slightly optimistic *p* values for the exact tests, although the large margins by which the key attitudinal items exceeded 50% agreement make it unlikely that our substantive conclusions would change. Because participation relied on open, non-probability recruitment, the estimates reported here should not be interpreted as population-prevalence measures for Croatian nurses or students. The magnitude of agreement levels—particularly the high proportions endorsing policy-related items—may therefore reflect characteristics of a self-selected, more policy-engaged subgroup.

Fifth, although we report internal consistency indices for the adapted domains, we did not perform exploratory or confirmatory factor analysis, item response modelling, or test–retest reliability for the Croatian version. Domain-level α values and item distributions should therefore be interpreted as basic diagnostics rather than proof of construct validity. Future work should undertake dedicated psychometric validation of Croatian political-competence scales, including dimensionality and stability over time. As a result, domain-level interpretations should be viewed as tentative indicators rather than validated constructs, and inferences about the relative strength of attitudes, knowledge, or skills domains should be interpreted with appropriate caution.

Lastly, our regression model adjusts only for age at professional start, years of experience, gender, and membership in professional or civic associations. We did not collect information on highest educational attainment, detailed workplace setting (e.g., hospital vs. primary care, public vs. private), or managerial role, so residual confounding by these factors is likely. Estimates should therefore be interpreted as partially adjusted associations, not as fully confounder-controlled causal effects.

### 4.5. Future Research

Confirmatory tests targeted item-level proxies rather than latent-scale scores; future work should validate Croatian domain scores for construct-level testing, and consider longitudinal or mixed-methods designs to test how educational interventions influence political confidence over time.

Three directions emerge:

1. Curricular evaluation: Test the impact of targeted political-competence modules within nursing programs.
2. Organizational culture: Examine how institutional hierarchy and managerial support affect nurses’ willingness to engage in policy.
3. Cross-national comparison: Benchmark Croatia’s findings against other Central and Eastern European countries to inform regional capacity-building initiatives.

## 5. Conclusions

This cross-sectional survey provides descriptive evidence that nurses and nursing students in Croatia express strong attitudinal support for the profession’s involvement in health-policy processes and consistently endorse the importance of scientific evidence in decision-making. At the same time, self-reported confidence in influencing policy remained moderate, with lower political self-efficacy particularly evident among older and more experienced respondents. This pattern indicates a distinction between valuing policy engagement conceptually and feeling personally equipped to participate in it.

These descriptive findings are consistent with several potential implications for education, professional development, and organizational practice. The coexistence of broad attitudinal readiness and uneven confidence suggests that future efforts to strengthen political competence may need to address both knowledge and evidence orientation as well as contextual and experiential factors that shape perceived self-efficacy. The age- and experience-related gradients observed in this study point to the relevance of organizational cultures, expectations, and opportunities for engagement across the career course.

From a policy and workforce perspective, the results highlight areas where clearer structures for participation, exposure to policy processes, and supportive professional environments may contribute to greater alignment between conceptual readiness and perceived capacity. While this study cannot determine the direction or magnitude of such relationships, it identifies concrete domains in which future research may be informative. These include evaluations of educational interventions, examinations of organizational determinants of political self-efficacy, and longitudinal or probability-based studies to assess how political competence develops over time.

By providing context-specific quantitative evidence from an understudied Central and South-Eastern European setting, this study contributes to the international literature on nurses’ political competence and offers a descriptive foundation for subsequent research, curricular planning, and policy dialogue in Croatia.

## Data Availability

The anonymized dataset of this study is openly available in the Open Science Framework (OSF) repository at https://doi.org/10.17605/OSF.IO/49NJS. The OSF repository contains a single XLSX file with the fully anonymized dataset. All variables are labeled with descriptive column names corresponding to the questionnaire items, which allows reconstruction of item wording using the questionnaire provided in the Supplementary Material. Direct identifiers (e.g., IP address, email, timestamps) were never collected by LimeSurvey, and no free-text fields were included. Only de-identified categorical or numeric responses are provided. No additional codebook or analysis scripts were uploaded, as all variable labels are embedded in the XLSX file.

## Supplementary Materials

The following supporting information can be downloaded at: https://www.mdpi.com/article/10.3390/nursrep15120448/s1, Figure S1: Adjusted odds ratios (aOR) from an ordinal logistic regression of self-assessed policy confidence (N = 390); Table S1: Internal consistency and item-level descriptive statistics; Table S2: Full response distributions for all 19 Likert items; Table S3: Wilcoxon signed-rank sensitivity tests versus neutral (score = 3); Table S4: Stratified percentages agreeing (≥4) for working conditions and ability to propose policy changes; Table S5: Generalized ordered-logit (VGAM) sensitivity analysis relaxing the proportional-odds assumption for association membership.

## Public Involvement Statement

No public involvement in any aspect of this research.

## Guidelines and Standards Statement

This manuscript was drafted against the CHERRIES (Checklist for Reporting Results of Internet E-Surveys) and STROBE (STrengthening the Reporting of OBservational studies in Epidemiology) guidelines for cross-sectional studies.
