# Supplementary material for: Political Competence and Evidence-Informed Policy Engagement Among Nurses and Nursing Students in Croatia: A Cross-Sectional Study
Source: Nurs Rep. 2025 Dec 14;15(12):448. doi: 10.3390/nursrep15120448 (PMC12736083; doi:10.3390/nursrep15120448)

Supplementary Table S1. Internal consistency and item-level descriptive statistics

1.1. Panel A. Domain-level reliability

domain	k (items)	n (respondents)	Cronbach's α
Attitudes	6	390	0.687
Knowledge	3	390	0.731
Skills	6	390	0.837

1.2. Panel B. Item-level descriptive statistics

domain	Item (statement)	n	mean	SD	median	Q1	Q3
Attitudes	Smatram da sestrinstvo ima utjecaj napraviti promjene određenih zdravstvenih politika	390	3.65	1.17	4	3	4.75
Attitudes	Znam da imam dovoljno utjecaja mijenjati zdravstvenu politiku ali kako taj utjecaj upotrijebiti	390	3.09	1.11	3	2	4.00
Attitudes	Smatram da sestrinstvo ima dobar položaj unutar zdravstvenog tima	390	2.47	1.22	2	2	4.00
Attitudes	Uvjeti rada utječu na sudjelovanje medicinskih sestara u zdravstvenim politikama	390	3.95	1.01	4	4	5.00
Attitudes	Vjerujem da je sestrinstvo ključan dio za promjenu zdravstvenih politika jer ima holističku viziju korisnika zdravstvenih usluga	390	4.08	0.93	4	4	5.00
Attitudes	Mislim da je korištenje znanstvenih dokaza ključan čimbenik pri donošenju zdravstvenih politika	390	3.84	1.04	4	3	5.00
Skills	Sposoban na sam primijeniti kritičko mišljenje kako bih izvršio/la promjenu u sestrinskoj praksi	390	3.72	0.97	4	3	4.00
Skills	Vjerujem da sam sposoban na utjecati na sliku koju ostalo zdravstveno osoblje ima o sestrinstvu	390	3.73	0.88	4	3	4.00
Skills	Osjećam se kvalificiranim utjecati na zdravstvene politike poznavajući uvjete rada i obrazovanja medicinskih sestara	390	3.40	1.06	4	3	4.00
Skills	Sposoban/na sam provoditi skrb usmjerenu na ljude i biti u stanju provoditi	390	3.81	0.96	4	3	4.00

domain	Item (statement)	n	mean	SD	median	Q1	Q3
	kroz to prijedloge za političke promjene u korist poboljšanja skrbi						
Skills	Osjećam se sposobnim/om formirati prijedloge za promjene u zdravstvenim politikama	390	3.24	1.12	3	2	4.00
Skills	Mogu primijeniti znanstvene dokaze kako bih počeo/la kreirati..političke promjene u svom radu	390	3.43	1.02	4	3	4.00
Knowledge	Znam da skrb usmjerena na dobrobit čovjeka omogućuje razinu individualizacije i kontekstualizacije politika zbog kojih sestrinstvo ima jedinstvenu viziju promjene	390	3.94	0.90	4	3	5.00
Knowledge	Znam da nedostatak samopouzdanja kao i nedostatak vještina sprječava sestrinstvo da se uključi u politiku	390	3.95	1.04	4	4	5.00
Knowledge	Shvaćam da su znanstveni dokazi jedan od temeljnih stupova za izgradnju sestrinstvo s većim obujmom sudjelovanja u zdravstvenim politikama	390	3.82	1.04	4	3	5.00

Items are presented in the original Croatian wording used in the survey.

Supplementary Table S2. Full response distributions for all 19 Likert items

Item	N	% 1 (Strongly disagree)	% 2 (Disagree)	% 3 (Neutral)	% 4 (Agree)	% 5 (Strongly agree)
Apply evidence for change	390	8.2	25.4	23.1	35.9	7.4
Care enables policy change	390	2.6	7.7	18.5	48.5	22.8
Confidence and skills barrier	390	3.3	15.4	30.5	36.9	13.8
Critical thinking for change	390	1.5	5.9	12.1	43.8	36.7
Evidence as pillar	390	1.8	9.2	17.4	56.9	14.6
Evidence key for policy	390	5.9	14.6	13.1	41.3	25.1
Formulate policy proposals	390	23.1	39.0	12.1	19.2	6.7
Holistic care guides change	390	3.3	10.5	12.8	47.4	25.9
Holistic perspective key	390	6.7	21.3	24.9	35.4	11.8
Influence team perception	390	3.3	9.5	8.5	46.7	32.1
Knowledge of leadership	413	7.5	21.8	32.9	27.6	10.2
Nurse creates change	413	2.2	4.6	9.7	27.1	56.4
Nurse in political decisions	413	3.1	6.5	15.0	24.2	51.1
Nursing impacts policies	390	4.4	16.7	27.4	37.4	14.1
Nursing should propose reforms	413	1.0	5.8	6.5	14.8	71.9
Nursing well-positioned in team	390	2.8	9.0	9.7	47.4	31.0
Policy influence ability	390	2.8	10.8	14.1	44.1	28.2
Qualified for policy influence	390	2.3	11.5	16.7	51.0	18.5
Working conditions matter	390	1.3	5.4	19.5	45.4	28.5

Supplementary Table S3. Wilcoxon signed-rank sensitivity tests vs neutral (score = 3)

Sentinel item	N	Median	Q1	Q3	Wilcoxon V	p-value
**H1:** *Working conditions affect nurses' policy participation*	390	4	4	5	55,109.0	< 0.001
**H2:** *Scientific evidence is key to nursing policy*	390	4	3	5	48,740.0	< 0.001
**H3:** *Nursing's holistic perspective is essential for reform*	390	4	4	5	55,139.5	< 0.001

Supplementary Table S4. Stratified %Agree (≥4) for working conditions and ability to propose policy changes

Panel A. Stratified by age group

Outcome	Age group	N	n (Agree ≥4)	% Agree
Ability to propose policy changes	18-24	111	57	51.4
Ability to propose policy changes	25-35	107	48	44.9
Ability to propose policy changes	36-45	86	36	41.9
Ability to propose policy changes	46-50	54	28	51.9
Ability to propose policy changes	50+	32	15	46.9
Working conditions → policy participation	18-24	111	97	87.4
Working conditions → policy participation	25-35	107	87	81.3
Working conditions → policy participation	36-45	86	57	66.3
Working conditions → policy participation	46-50	54	40	74.1
Working conditions → policy participation	50+	32	25	78.1

Panel B. Stratified by association membership

Outcome	Membership	N	n (Agree ≥4)	% Agree
Ability to propose policy changes	no	178	99	55.6
Ability to propose policy changes	yes	212	85	40.1
Working conditions → policy participation	no	178	156	87.6
Working conditions → policy participation	yes	212	150	70.8

Supplementary Table S5. Generalized ordered-logit (VGAM) sensitivity analysis relaxing the proportional-odds assumption for association membership

Panel A. Predictors with proportional odds (parallel slopes)

Predictor	Adjusted odds ratio (aOR)	95% CI	*p*-value
Work experience (per year)	1.01	0.99–1.03	0.169
Age at professional start (per year)	0.95	0.91–0.99	0.017
Female vs male	1.00	0.6–1.67	0.988

Panel B. Membership effects allowing non-parallel odds

Cumulative threshold	Adjusted odds ratio (aOR) (membership yes vs no)	95% CI	p-value
P(Y ≥ 2 vs 1)	15.42	7.87–30.2	< 0.001
P(Y ≥ 3 vs 1–2)	2.20	1.42–3.4	< 0.001
P(Y ≥ 4 vs 1–3)	0.91	0.6–1.38	0.647
P(Y = 5 vs 1–4)	0.21	0.13–0.33	< 0.001

Note: Cumulative thresholds represent generalized ordered-logit contrasts comparing the odds of reporting responses at or above a given category versus lower categories (e.g., $P(Y \geqslant 3)$ vs $P(Y \leqslant 2)$).

Figure S1 Adjusted odds ratios (aOR) with 95% CIs from an ordinal logistic regression of self-assessed policy confidence (N = 390). The dashed line denotes aOR = 1. Predictors: years of experience, age at professional start, gender, and association membership. Brant testing indicated non-proportional odds for membership; a generalized ordered-logit sensitivity (VGAM) did not change conclusions (Table S5).

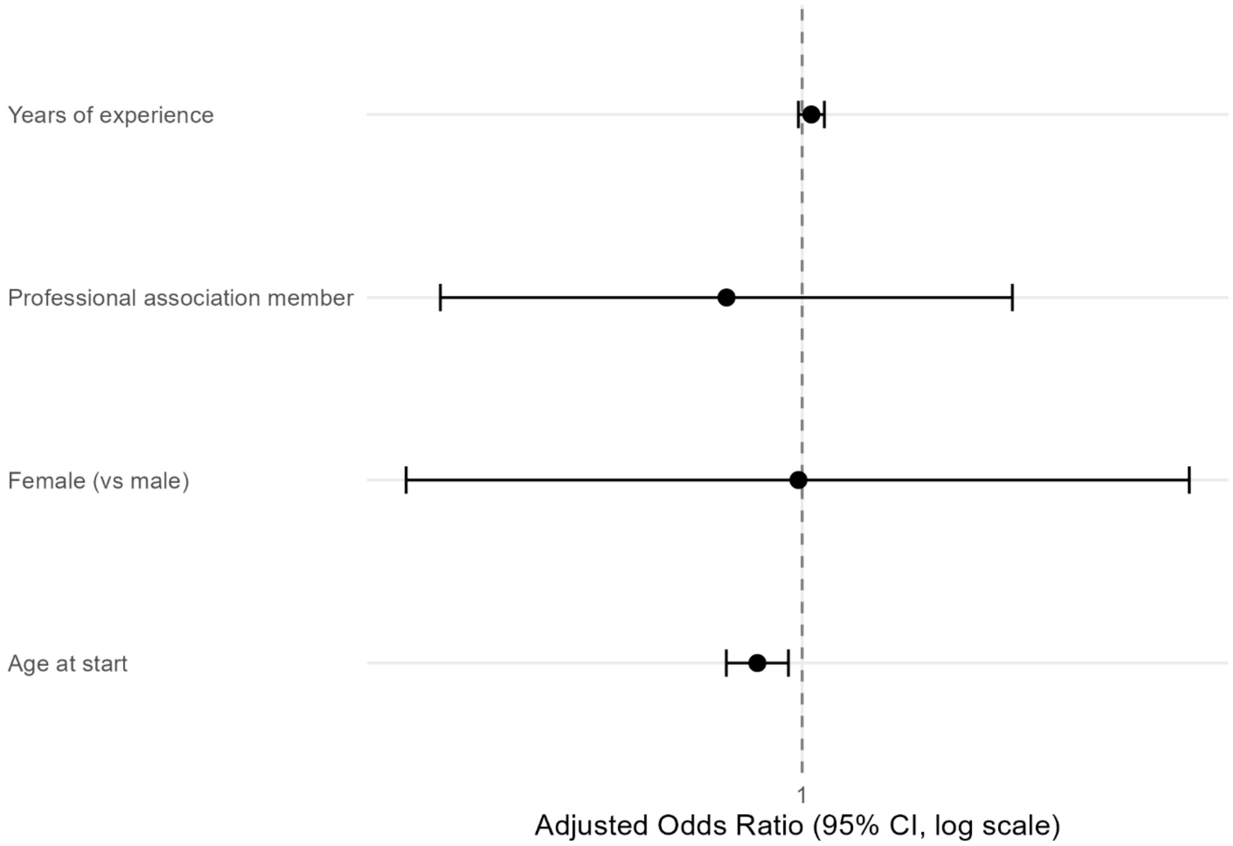

Supplement: Supplementary file 1 [file nursrep-15-00448-s001.zip › nursrep-3979603-supplementary.pdf]
